# Supplementary figures and images for: Large-scale characterization of drug mechanism of action using proteome-wide thermal shift assays
Source: eLife. 2024 Nov 11;13:RP95595. doi: 10.7554/eLife.95595 (PMC11554310; doi:10.7554/eLife.95595)

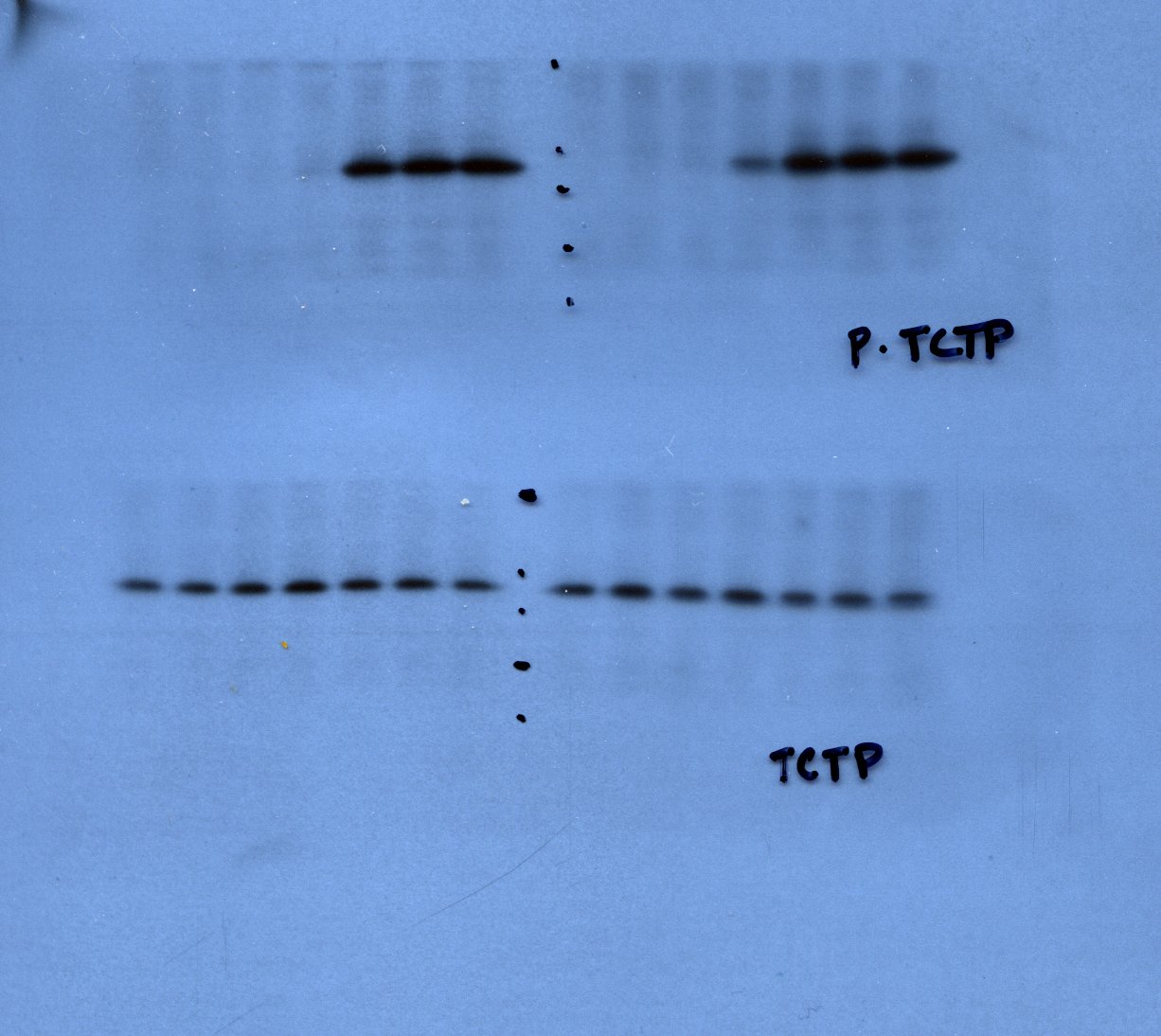

Supplement: Figure 2—source data 4. [file elife-95595-fig2-data4.zip › Figure 2 - source data 4/Figure 2 - source data 4 - TCTP_p-TCTP.jpg]

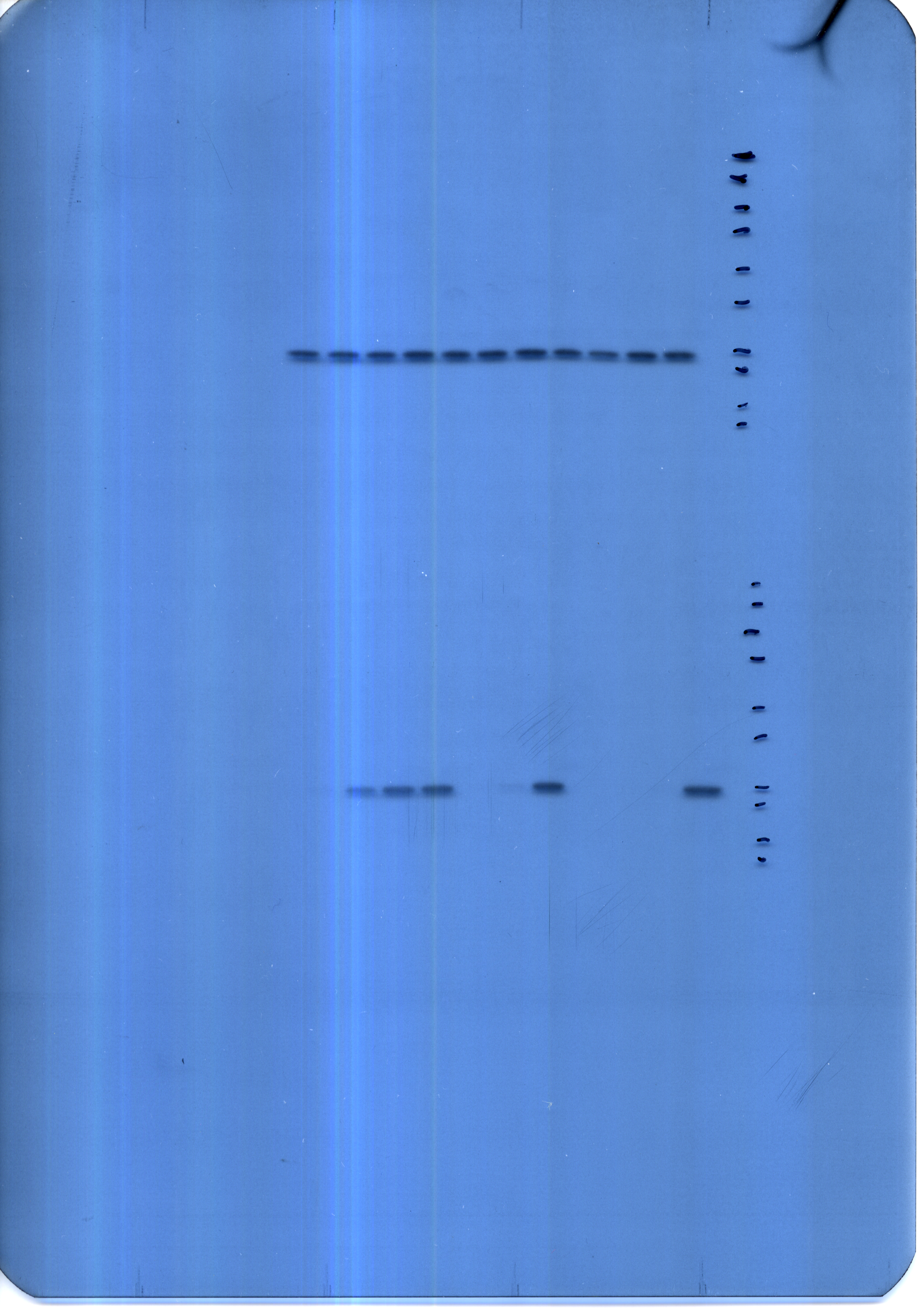

Supplement: Figure 2—source data 6. [file elife-95595-fig2-data6.zip › Figure 2 - source data 6/Figure 2 - source data 6 - TCTP_p-TCTP.tif]

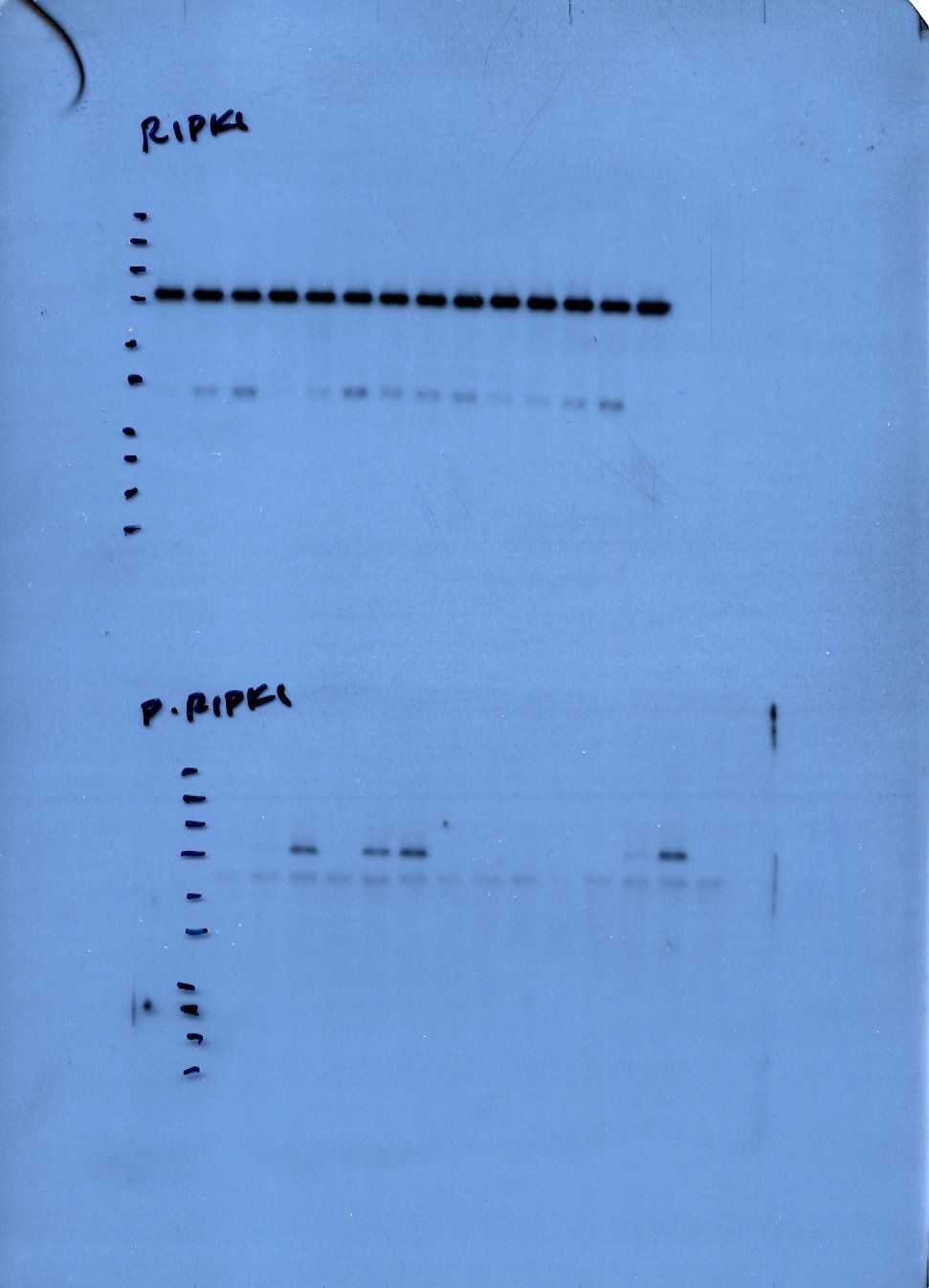

Supplement: Figure 4—source data 3. [file elife-95595-fig4-data3.zip › Figure 4 - source data 3/Figure 4 - source data 3 - p-RIPK1.jpg]

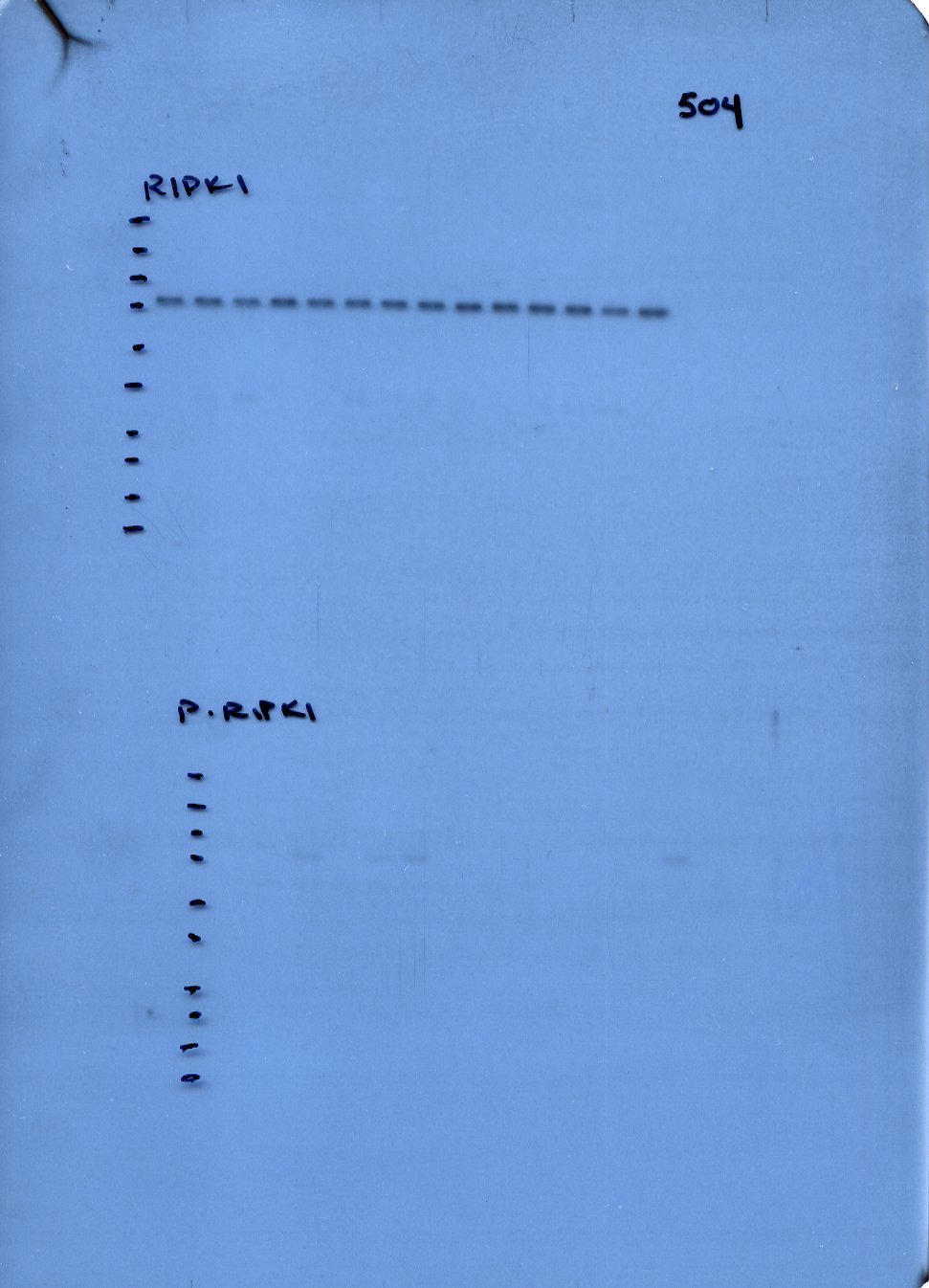

Supplement: Figure 4—source data 3. [file elife-95595-fig4-data3.zip › Figure 4 - source data 3/Figure 4 - source data 3 - RIPK1.jpg]

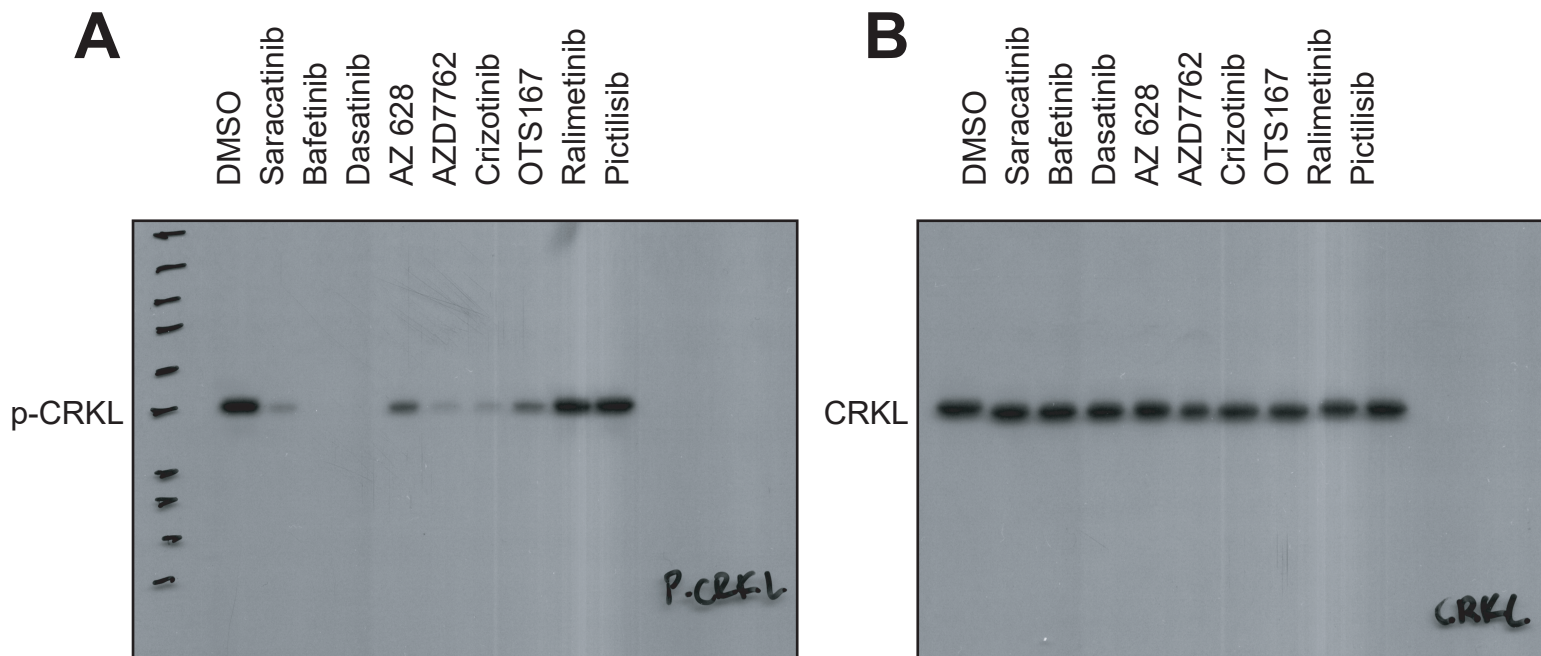

Figure 5C. Unedited scans of pCRKL (A) and CRKL (B).

Supplement: Figure 5—source data 1. [file elife-95595-fig5-data1.zip › Figure 5 - source data 1/Figure 5 - source data 1.pdf]

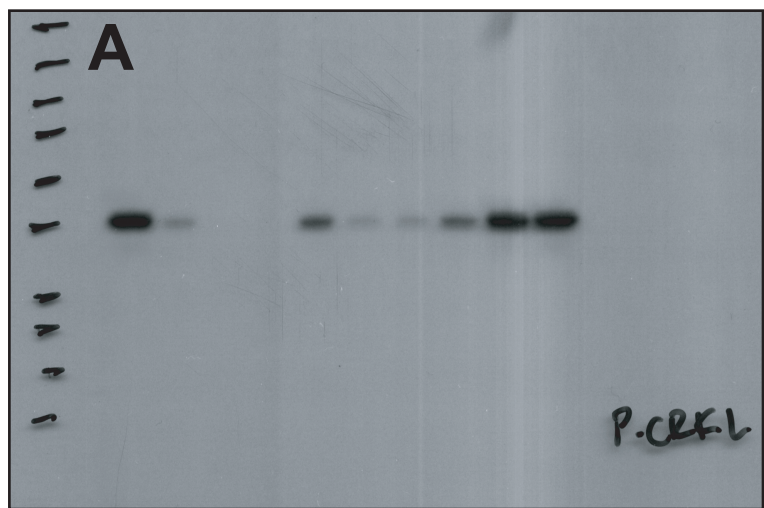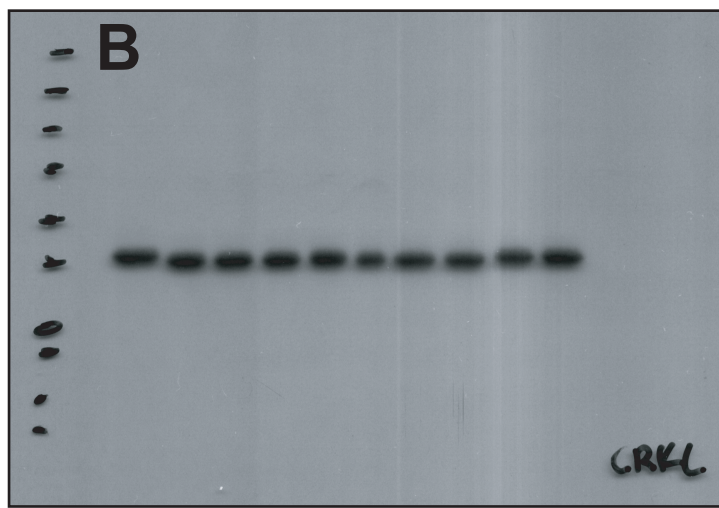

Figure 5C. Unedited scans of pCRKL (A) and CRKL (B).

Figure 5 - source data 1

Supplement: Figure 5—source data 2. [file elife-95595-fig5-data2.pdf]
