# Supplementary material for: Large-scale characterization of drug mechanism of action using proteome-wide thermal shift assays
Source: eLife. 2024 Nov 11;13:RP95595. doi: 10.7554/eLife.95595 (PMC11554310; doi:10.7554/eLife.95595)
Supplement: Figure 2—source data 5. [file elife-95595-fig2-data5.zip › Figure 2 - source data 5/Figure 2 - source data 5.pdf]

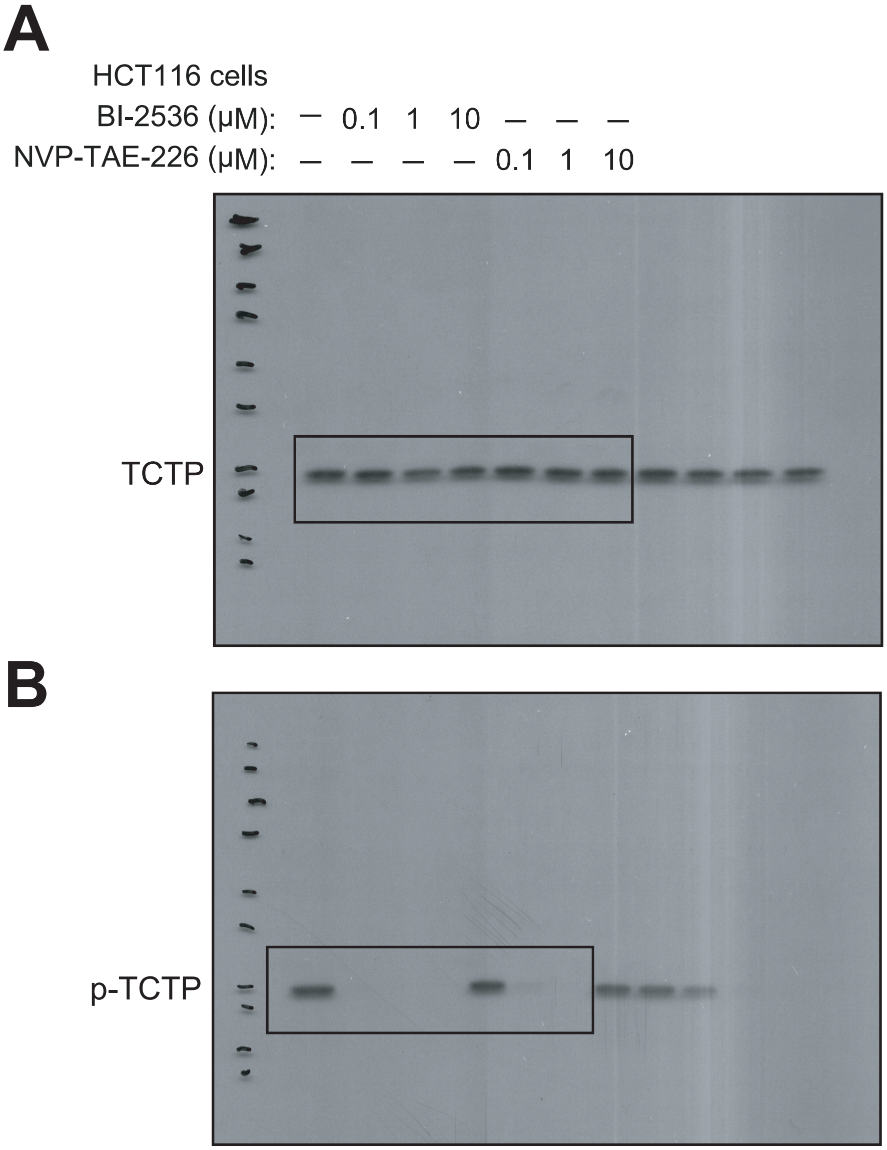

Figure 2 - figure supplement 1D. Unedited scans of TCTP (A) and pTCTP (B).
