# Supplementary material for: Large-scale characterization of drug mechanism of action using proteome-wide thermal shift assays
Source: eLife. 2024 Nov 11;13:RP95595. doi: 10.7554/eLife.95595 (PMC11554310; doi:10.7554/eLife.95595)
Supplement: Figure 4—source data 2. [file elife-95595-fig4-data2.zip › Figure 4 - source data 2/Figure 4G.pdf]

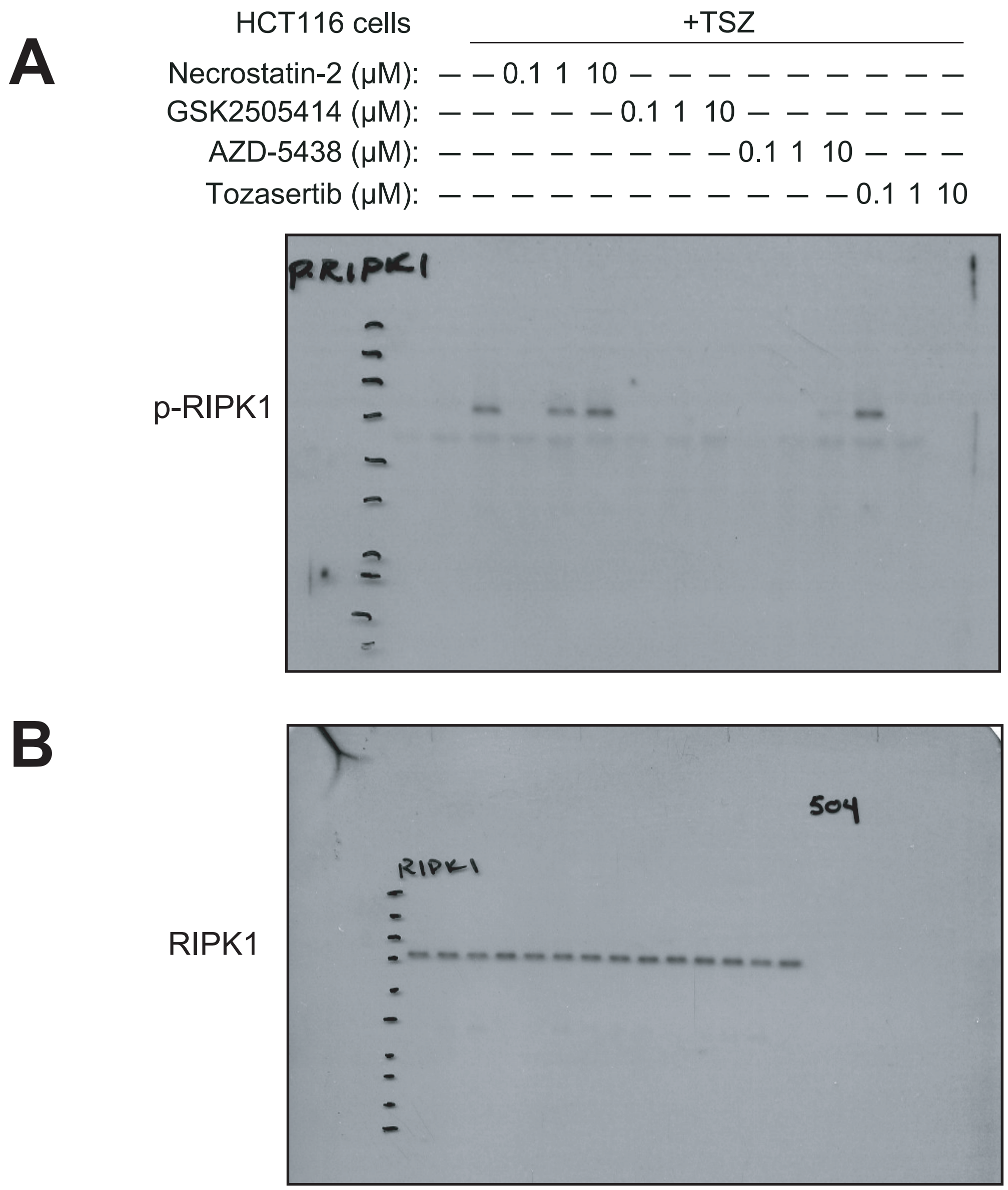

Figure 4G. Unedited scans of pRIPK1 (A) and RIPK1 (B).
